# An Engineered Specificity of Anti-Neoplastic Agent Loaded Magnetic Nanoparticles for the Treatment of Breast Cancer

**DOI:** 10.3390/polym13213623

**Published:** 2021-10-20

**Authors:** Anroop B. Nair, Mallikarjun Telsang, Riyaz Ali Osmani

**Affiliations:** 1 Department of Pharmaceutical Sciences, College of Clinical Pharmacy, King Faisal University, Al-Ahsa 31982, Saudi Arabia; 2 Department of Surgery, College of Medicine, King Faisal University, Al-Ahsa 31982, Saudi Arabia; mvtelsang@kfu.edu.sa; 3 Nanomedicine Lab, Department of Biosciences and Bioengineering, Indian Institute of Technology Bombay (IIT-B), Mumbai 400076, India; riyazosmani@gmail.com

**Keywords:** magnetic nanoparticles, gemcitabine, Box–Behnken design, cancer cell lines

## Abstract

Nanoparticles have gained increased attention due to the prospection of drug delivery at target sites, thus limiting the systemic effects of the drugs. Their efficiency was further improved by adding special carriers such as magnetite (Fe_3_O_4_). It is one of the extensively used oxides of iron for both pharmaceutical and biomedical applications owing to its ease of preparation and biocompatibility. In this work, Gemcitabine magnetic nanoparticles were prepared using Fe_3_O_4_ and chitosan as the primary ingredients. Optimization was accomplished by Box–Behnken Design and factor interactions were evaluated. The desirability function approach was made to enhance the formulation concerning particle size, polydispersity index, and zeta potential. Based on this, optimized magnetic nanoparticles (O-MNP) were formulated with 300 mg of Fe_3_O_4_, 297.7 mg of chitosan, and a sonication time of 2.4 h, which can achieve the prerequisites of the target formulation. All other in vitro parameters were found to be following the requirement. In vitro cytotoxic studies for O-MNP were performed using cell cultures of breast cancer (MCF-7), leukemia (THP-1), prostate cancer (PC-3), and lung cancer (A549). O-MNP showed maximum inhibition growth with MCF-7 cell lines rather than other cell lines. The data observed here demonstrates the potential of magnetic nanoparticles of gemcitabine in treating breast cancers.

## 1. Introduction

Breast cancer is widespread among women in a great number of countries across the globe [1]. Indeed, even with the rigorous attempts within the last five decades, the success rate in cancer therapy seems to be marginal and could not significantly reduce cancer mortality. Alternatively, significant advances have been accomplished for the other ailments, as well as the cardio, pulmonary, and cerebrovascular diseases [2]. Currently, the essential methodologies for cancer therapy include surgery, radiation, and chemotherapy. However, in any case, these methodologies may impose potential adverse effects that are not ample for remedial management of advanced or metastasized cancers. Moreover, the protective immunoglobulin antibodies are being employed progressively in oncology, albeit the antibody treatment is often expensive and sometimes has undesirable systemic effects [3]. Nonetheless, the consent of some preceding signs for the antibody treatment has even been removed in recent times, for the treatment of colorectal cancer [4]. There have not yet been genuine forward leaps in the treatment of cancer, by and large. The traditional agents used for chemotherapy in oncology drug development still manifest significantly fewer outcomes in getting up to target tumor location and are often constrained by dose-limiting toxicity. By integrating both controlled release technology and targeted smart drug delivery systems for the development drug formulation may furnish a better effective and less deleterious way out to overcome constraints in traditional chemotherapy.

Tumors possess distinct physiological features which allow them to resist traditional treatment approaches. This, combined with the complexity of the biological system, presents significant hurdles to the site-specific delivery of therapeutic drugs. Current clinical approaches are based on the systemic administration of chemotherapeutics drugs. These therapies are limited by solubility and pharmacokinetic factors on account of their physicochemical properties, as well as fraught with toxicity issues as they generally target any rapidly dividing cells in the body such as those of the hair, skin, spleen, and liver, among others. Therefore, delivery of these anti-neoplastic agents with the use of nanoparticles (NPs) helps overcome some of these disastrous side effects. As indicated by the appraisals by the US National Cancer Institute, nanomedicine will end up being explored later on avoidance, diagnostic examination, and therapy of malignancy [5]. Nanotechnology has effectively discovered utilizations in numerous clinical fortes, for instance, in otorhinolaryngology [6]. It is generally utilized in various orders, ranging from clinical images to regenerative medication. The appreciation of nanoparticle technology and innovation has increased enormously in various fields, among which biomedicine has applications that are close to today’s life [7,8,9].

Recent investigations are exploring to establish targeted curative methods by using outside forces, together with electromagnetic fields, light, ultrasound, temperature, and mechanical forces to improve drug concentration within cancer locations [10,11,12]. In this method, the drug substance is restricted to a particular targeted site through superficially created magnetization. By appliance of peripheral magnetization towards where the drug is progressively discharged, the magnetic components get linked with drug molecules and aimed to exact sites of the body, thus convalescing the therapeutic drug competence by decreasing the auxiliary toxic effects on the allied healthy normal cells or tissues [13].

Few examinations named superparamagnetic iron oxide nanoparticles (SPIONs) have been anticipated to be applied for ailment diagnosis and therapy (or theranostics), especially on account of strong tumors [14,15,16,17]. It is unequivocally their reaction to one or the other direct current or alternate current magnetic fields which determines the applications of magnetic nanoparticles (MNPs) in biomedical fields. Drug conveyance being one of them; the particles were important for alleged attractive vectors, that are stacked alongside a chemotherapeutic drug and functionalize so they generally can evade from the immune system, as well as having the option to explicitly connect to target tumor cells. A non-homogeneous remotely adapted magnetic force may be utilized to direct the formulated magnetic nano systems to the site of action or, at any rate, retain them in the wake of being shipped by the circulatory system [18,19,20,21].

Chitosan (CS) is a polymeric compound acquired through chitin deacetylation. This polymer is degradable, innocuous, economical, and possesses a high absorption potential, environment compatibility, rapid kinetics, is highly efficient in eliminating an extensive set of dyes and metals, and is feasible in providing numerous derivatives. Additionally, CS was found to be a promising nanocarrier of anti-neoplastic drugs in many reports [22]. Furthermore, the principal amino groups of CS can be utilized for designing controlled release, mucoadhesion, in situ gelling, transfection delivery, penetration enhancement, and hampering efflux pumps [23,24,25]. CS can effortlessly affix to the magnetic nanoparticle surface and also render the amine and hydroxyl groups for coupling with the therapeutic drug molecules [26]. CS nanocarriers consisting of magnetite (Fe_3_O_4_, iron oxide) core, can be effectively directed to the tumor site by applying external magnetic force subsequently following the anti-tumor drug load. It is an important benefit of operating MNPs. An additional advantage of operating MNPs is their function in tumor imaging as MRI agents, which was endorsed for clinical use by FDA [27].

Gemcitabine (2′,2′-difluorodeoxycytidine) is selected as an anti-neoplastic agent, which is a nucleoside analogue that was found to possess the antitumor activity and also have established reports for various types of human tumors, inclusive of breast cancer for both experimental as well as in clinical studies [28,29]. Gemcitabine impedes the synthesis of DNA by integrating it into DNA strands. As a result, DNA polymerase fails to add on nucleotides, which lead to the cessation of chain elongation and promote cell death [30]. Nevertheless, Gemcitabine may give rise to considerable systemic toxicities and resistance issues, which limits its therapeutic efficiency [31]. The plasma concentration of the drug may rapidly dip under the viable threshold degree because of the less elimination half-life (8–17 min) and also restrict its clinical benefit. In this manner, a lot bigger dosages are needed to arrive at efficacious plasma levels, which may increase the possibility of side effects.

Few studies in the literature have been carried out on the development of Gemcitabine delivery system using nanoparticles that could reduce its side effects, increase internalization of the drug without receptor mediation, and prolong its retention time [32,33,34,35]. Yet, few researchers worked on magnetic nanoparticles of Gemcitabine using polyhydroxy butyrate and chitosan coatings [36]. Chitosan has gained considerable attention due to its biocompatibility, biodegradability, and non-toxicity. Chitosan-based delivery systems are widely used for the controlled delivery of drugs, proteins, and peptides [37]. Owing to the aforesaid reasons, this study considered the advantage of the compatibility and innocuous characteristics of CS and aimed to optimize various formulation parameters effectively to enhance the anticancer activity. The Gemcitabine coupled Fe_3_O_4_@CS nanoparticles were further examined for anti-cancer properties.

## 2. Materials and Methods

### 2.1. Materials

Gemcitabine was obtained from Intas Pharm, Ahmedabad, India. CS was purchased from High Media Lab, Mumbai, India. Fe_3_O_4_ was procured from S.D Fine Chemicals, Mumbai, India. All the remaining chemicals and solvents that were used are of analytical grade. For all the experiments we used freshly obtained double distilled water. Breast cancer cell lines (MCF-7), leukemia cancer cell lines (THP-1), prostate cancer cell lines (PC-3), and lung cancer cell lines (A549) were procured from National Centre for Cell Science, Pune, India.

### 2.2. ATR-FTIR Characterization

The Fourier-transform infrared radiation (FTIR) spectral readings were noted at environment temperature by employing attenuated total reflection mode (ATR-FTIR) in a JASCO 6200 FT-IR (Tokyo, Japan) spectrometer with SPECTRA MANAGER V2 software (JASCO, Tokyo, Japan). The test sample was examined without advance conduct at ambient temperature with fifty scans and a 4 cm resolution [38].

### 2.3. Preparation of the Magnetic Fe_3_O_4_ Nanoparticles

MNPs were prepared by simultaneous precipitation of the Fe^+3^ and Fe^+2^ ions with 2:1 molar proportion, in the incidence of ammonium hydroxide [NH_4_OH]. To be precise, a 100 mL aqueous solution with 0.0216 M FeCl_3_.6H_2_O and 0.0108 mol FeCl_2_.4H_2_O was made and subjected for heating at 85 °C with N_2_ air. The mixture was briskly agitated at 500 rpm. Subsequently, 10 mL of 25% NH_4_OH was gradually introduced in a single shot into the Fe ion solution. The introduction of aqueous NH_3_ led to the instantaneous development of the black MNPs. Further, the solution was incessantly agitated for 25 min and settled to cool to room temperature [24,39]. Later, the dark MNPs were segregated magnetically out of the solution and cleaned thrice with deionized water.

### 2.4. Synthesis of Fe_3_O_4_ NP@CS (Fe_3_O_4_@chitosan)

Initially, 50 mL of acetic acid solution (2% *v*/*v*) consisting of 0.25 g CS (98% NH_2_) was made and agitated for 2 h. Further, 500 mg of the synthesized MNPs was suspended in 50 mL of the prepared CS solution (CS to Fe_3_O_4_ weight ratio = 1:2). Later the acquired mixture was elicited to 150 mL (to get homogenous mixture and for easy mixing), agitated (1 h) at ambient temperature, and counterbalanced with a 10% *w*/*v* NaHCO_3_ solution. The resulting particles (Fe_3_O_4_@CS) were separated by applying an external magnetic field, cleaned consequently using distilled water and ethyl alcohol, and further desiccated at normal temperature under a vacuum environment.

### 2.5. Preparation of Fe_3_O_4_@CS/Gemcitabine

To load Gemcitabine onto the Fe_3_O_4_@CS nanoparticles, 0.05 g of the Fe_3_O_4_@CS nanoparticles were mixed with 30 mL of an aqueous solution of Gemcitabine (2.5 mM). The mixture was agitated continuously for 1 day at room temperature. Sonication was performed by an ultrasonic bath with a power of 13 W and >20 kHz frequency. During the process, the solution pH was tuned to 7.4. The obtained solid output was segregated by applying an external magnetic field, cleaned with distilled water, and desiccated at normal temperature under a vacuum environment to achieve the end Fe_3_O_4_@CS/Gemcitabine product.

### 2.6. Optimization of Fe_3_O_4_@CS MNP

Preparation of Fe_3_O_4_@CS MNP was enhanced statistically through RSM (Response Surface Methodology). This method aids in identifying the (a) premium processing condition; (b) important elements and their interactivity through few experimental conduct [40]. Selected sovereign variables were the concentration of Fe_3_O_4_ (X_1_), CS conc. (X_2_) and sonication time (X_3_) at three various stages, were encoded as low (−1), medium (0), and high (+1). These elements were optimized for particle size (PS-Y_1_), polydispersity index (PDI-Y_2_), and zeta potential (Y_3_). Box–Behnken design was employed using Design Expert 12 (Stat Ease Inc., Minneapolis, MN, USA), which generates seventeen experimental trials. The Box–Behnken design is an independent quadratic design in that it does not contain an embedded factorial or fractional factorial design. In this design, the treatment combinations are at the midpoints of the edges of the process space and the center. These designs are rotatable (or near rotatable) and require 3 levels of each factor. The designs have limited capability for orthogonal blocking compared to the central composite designs. Table 1 shows a complete plan of experiment interns of coded and real values of selected variables and restraints of dependent variables. Statistical authentication of developed polynomial equations was performed through analysis of variance (ANOVA). Entire trials conducted were applied to various statistical designs (like the model, 2FI and quadratic, etc.) and the ideal model was chosen by comparing different statistical variables such as coefficient of variation (CV), multiple correlation coefficient (R^2^), and adjusted, predicted R^2^ values [41] to estimate the response in each trial. A quadratic model was utilized and regression analysis was also performed.

(1) $$Y_{i\left(\mathrm{Quadratic}\right)}=b_{0}+b_{1}X_{1}+b_{2}X_{2}+b_{3}X_{3}+b_{4}X_{1}X_{2}+b_{5}X_{1}X_{3}+b_{6}X_{2}X_{3}+b_{7}X_{1}^{2}+b_{8}X_{2}^{2}+b_{9}X_{2}^{3}$$

where Yi is the dependent variable; b_0_ is the arithmetic response of experimental trails; b_i_ is the estimated coefficient for independent variables X_1_, X_2_, and X_3_ (Main effects); X_1_X_2_, X_1_X_3_, and X_2_X_3_ correspond to the interaction terms and X_1_^2^, X_2_^2^, and X_3_^2^ to the polynomial terms.

### 2.7. Characterization

#### 2.7.1. Particle Size and Distribution

The MNPs were scattered in demineralized water and exposed to PS, size dissemination, and PDI quantification using a Malvern particle size analyzer (MS2000, Worcestershire, UK) [42].

#### 2.7.2. Estimation of Surface Charge

The synthesized magnetic drug nanoparticle zeta potential was measured by suspending the formulation with deionized water and then measurement was carried out with Malvern Zetasizer Nano ZS (MAL 000967, Worcestershire, UK) [43].

#### 2.7.3. Rationale of Experimental Design

An optimal stalk of optimized magnetic nanoparticles (O-MNP) was made with a streamlined concentration of individualistic factors and evaluated [44]. The optimum output of the experimental pattern can be confirmed by determining relative error through weighing up the predicted results with practical results as given in the equation below. O-MNP was prepared with the predicted values for further studies.

Relative error (%) = [(Predicted value − Practical value)/Predicted value] ×100(2)

### 2.8. Drug Loading and Surface Binding

Estimation of loading competence and surface binding of magnetic drug nanoparticles was done separating Fe_3_O_4_ arising out of nanoparticles. Momentarily, 2 mg of MNP was blended with 50 mL of methyl alcohol and permitted 20 min under sonication [45]. The subsequent blend was subjected to centrifugation for 10 min at a rate of 5000 rpm. The buoyant sample was analyzed using UV-Vis Spectrometer (Shimadzu 1800, Kyoto, Japan) at 269 nm. The drug loading efficiency and drug surface binding were resolved as follows [29].

Drug Loading Efficiency (%*w/w*) = Mass of the drug in Nanoparticles × 100 (3)

Mass of Nanoparticles surface binding (%*w/w*) = (Mass of the drug in Nanoparticles/ Mass of Nanoparticles) × 100 (4)

### 2.9. Scanning Electron Microscopy (SEM)

SEM was employed to estimate the surface characteristics and shape of the produced nanoparticles. Samples were coated with gold and placed on the sample container and pictures were captured (JEOL, JSM-6100, Tokyo, Japan) [46].

### 2.10. Magnetization Measurement

Magnetic hysteresis loops of both magnetized iron oxide nanoparticle and drug stacked nanoparticles were estimated with a vibrating sample magnetometer (7410, VSM, Lake Shore Cryotronics, Inc, Westerville, OH, USA) for the electromotive power initiated by magnetic particles in which the particles vibrate at a steady frequency and consistent magnetic field. The magnetic vulnerability of the prepared MNPs was solved utilizing the Fugro magnetic susceptibility meter.

### 2.11. In-Vitro Drug Release Profile

Dialysis sacks (cut-off size of 12–14 kDa) were loaded up with a pre-established measure of 2 mg each formulation that is kept in 40 mL of pH = 7.4 phosphate buffer solution utilized as receptor phase [47]. The mixture was blended and temperature-controlled at 37 °C. At a particular time-lapse, 2 mL of the receptor phase was collected and replaced with a fresh buffer. The quantity of Gemcitabine in the sample was estimated by calculating the absorbance of the supernatant spectrophotometrically at 269 nm in discrete time intervals. The aggregate amount of percentage drug release was enumerated and the graph was plotted against time [48].

### 2.12. In Vitro Cytotoxicity Studies

The anti-neoplasticactivity of O-MNP was performed using four different cell lines such as breast cancer cell lines (MCF-7- density of 15,000 viable cells/mL), leukemia cancer cell lines (THP-1-1.0 × 10^5^ to 1.5 × 10^6^ cells/mL), prostate cancer cell lines (PC-3; 3.0 × 10^4^ cells/cm^2^), and lung cancer cell lines (A549; 6 × 10^4^ cells/cm^2^).

#### 2.12.1. Cell Culture

Stock solutions of hydroalcoholic, alcoholic, and aqueous extracts were prepared (20 mg/mL) with DMSO (dimethyl sulfoxide): water (1:1) and then diluted with RPMI-1640/DMEM growth medium to get the desired concentrations. The stock solutions of chloroform, butanol, and hexane were prepared with DMSO, whereas the aqueous portion was added with distilled water. The cells were developed in a tissue culture flask when the cells are at a subconfluent stage, subsequently harvested with 0.05% trypsin in PBS solution consisting of 0.02% EDTA and draped in the medium. Cell suspension of 100 µL was grown in a 96-well tissue culture plate and then incubated for 24 h. Cells with at least 97% viability were only used to determine the cytotoxicity.

#### 2.12.2. Cell Treatment

The test samples were added to the wells of the culture plate and further incubated for 48 h. The cell growth was terminated by adding 50 µL of 50% of trichloroacetic acid and incubated for 1 h at 48 °C. Liquids were discarded, cleaned with distilled water, and then dried in the air. In the final step, sulforhodamine B dye (SBD) was added to all well plates and incubated for 30 min. The unbound SBD was eliminated by cleaning with 1% acetic acid and then dried. Bound SBD was dissolved by adding a tris-HCL buffer and the optical density was measured on ELISA at 540 nm [49].

Cancer cells only with DMSO (without any drug) and the conventional drug (epirubicin for treating breast cancers) served as control and positive control groups. The anti-neoplastic activity of O-MNP formulation containing Gemcitabine was compared with test and control at various concentrations such as 25, 50, 75, and 100 µL.

## 3. Results and Discussion

### 3.1. Fourier-Transform Infrared Radiation

**Figure 1:**
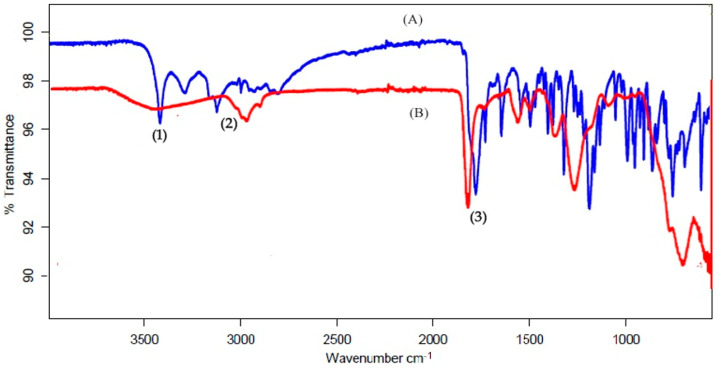
The FTIR spectra of (A) Gemcitabine and (B) physical mixture of Gemcitabine + all formulation ingredients. (1, 2 and 3-Characteristics peaks identified in A and B).

Figure 1, shows infrared spectra of Gemcitabine and physical mixture of Gemcitabine with all formulation ingredients. The spectra of Gemcitabine showed characteristic absorption peaks at 1728, 1065, and 633 cm^−1^ and others in the fingerprint region. Additionally, an intense band was present at 3410 cm^−1^ (in Figure 1) and 3010 cm^−1^ corresponding to the N–H stretching. The physical mixture of Gemcitabine with all the formulation ingredients does not cause any shift in the position of the Gemcitabine absorption bands, as the characteristic peaks were found to be in the same range. This confirms the absence of interaction between Gemcitabine and selected excipients.

### 3.2. Optimization of Fe_3_O_4_@CS MNP

The Box–Behnken design was used along with response surface methodology to optimize the preparation of Fe_3_O_4_@CS MNP by analyzing the impact of selected variables resulting in minimum PS, PDI, and maximum zeta potential. Seventeen runs were projected and their corresponding results were given in Table 2. Prepared nanoparticles possess hydrodynamic size ranges between 45 to 145 nm and a PDI of 0.26 to 0.47. Yet, another important parameter, the zeta potential of all the formulations were found to be 12–38 mV, which indirectly measures the stability of the formulations [50].

All the obtained results were analyzed using ANOVA and fx models to measure the individual responses and their effect on the variables. Based on F and p values obtained from the fit summary and the sequential sum of squares, a quadratic model was selected for the responses as the model is not aliased (Table 3) [41,51]. The sequential *p*-value for PS, PDI, and zeta potential was found to be <0.0001, 0.0002, and <0.0001, respectively. However, the lack of fit *p*-value was found to be non-significant proving the model was fit. Additionally, the data showed a difference of <0.2 between adjusted and predicted R^2^ values, implicating the fitness of the selected design. Design space can be navigated if the adeq. precision value is above four. The precision of the three responses was found to be 31.552, 20.4276, and 15.9408 concluding the suitability of the selected design and model. The high model F-value implies the model is significant.

**Figure 2:**
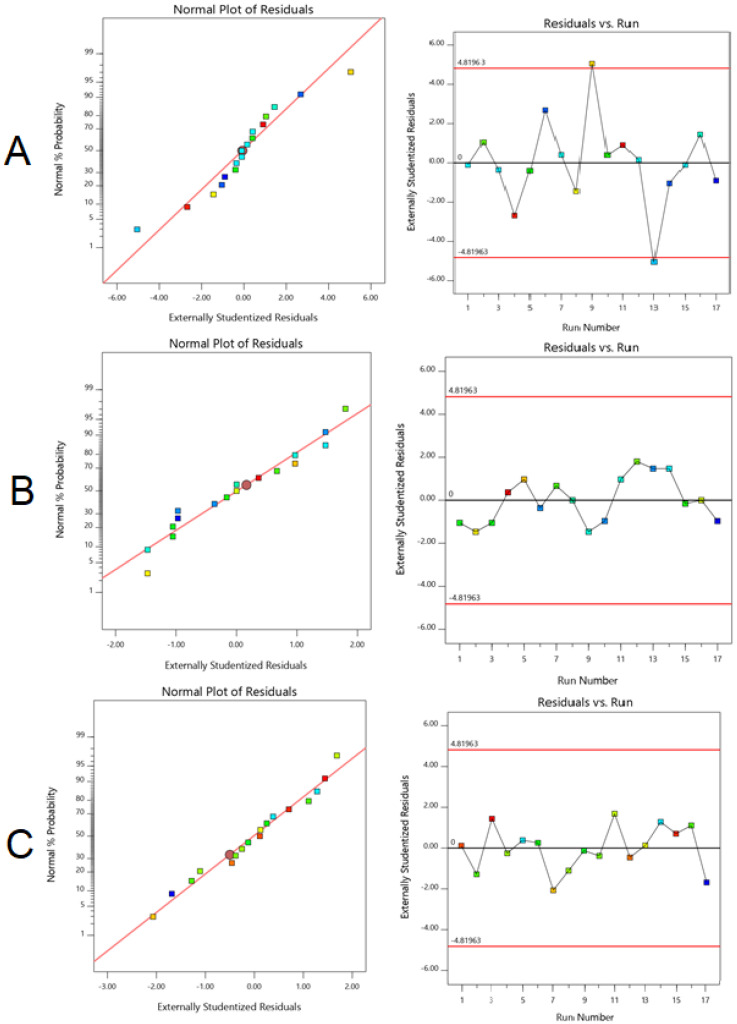
Normal probability plots of the residuals and residual models vs. test orders for all: (**A**) particle size, (**B**) polydispersity index, and (**C**) zeta potential.

Normal percentage probability and studentized residuals were plots additionally to quantify and validate the accuracy of models. This plot of residuals follows the normal distribution, with little deviation indicating the selected model was accepted statistically. Random scatter plots were observed in Figure 2, representing model residuals versus experimental runs, indicating the time-coupled variable slink in the background.

ANOVA was utilized to estimate the inference of factors by applying multiple regression to generate polynomial equations. The sequential and lack of fit values for all the responses are shown in Table 3 [39,40]. ANOVA results outraged the statistical significance of quadratic equations and the significance of model terms.

The experimental results indicated that PS is significantly affected by almost all actors except C and AB. Concerned *p* values were found to be <0.05 (Table 4). Coded equations can be helpful to predict the response for any given level of each factor and also identify the relative impact of the factors by comparing the factor coefficients. Except for the AC factor, all the rest are shown synergistic action, with the highest magnitude for factor A. The experimental design indicates that the PDI was potentially affected by (a) synergistic effect of B and C2 and (b) antagonist effect factor BC and polynomial terms of A and B. Zeta potential was affected significantly by A, BC (synergism), and factor C, all polynomial terms (antagonistic). The coefficient table of ANOVA and the generated regression equations confirm the great impact of Fe_3_O_4_ and CS on the formation and stability aspects of prepared MNP.

Particle size = + 69.40 + 36.37 A + 11.75 B − 11.63 C + 0.2500 AB − 0.5000 AC + 8.75 BC + 17.30 A² + 8.05 B² + 10.80 C²(5)

Polydispersity index = + 0.3720 + 0.0237 A + 0.0000 B − 0.0638 C − 0.0075 AB − 0.0050 AC − 0.0025 BC − 0.0310 A² − 0.0435 B² + 0.0340 C²(6)

Zeta potential = + 35.80 + 2.12 A + 5.50 B − 0.1250 C − 3.25 AB + 0.5000 AC +0.2500 BC − 3.40 A² − 8.15 B² − 4.90 C²(7)

**Figure 3:**
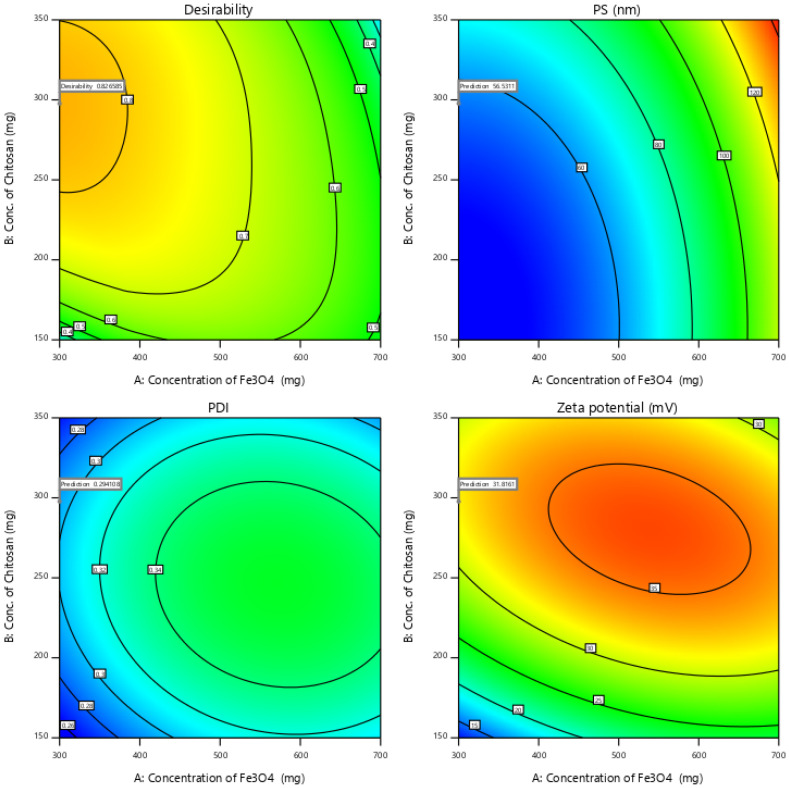
Desirability contour plots for all the responses.

**Figure 4:**
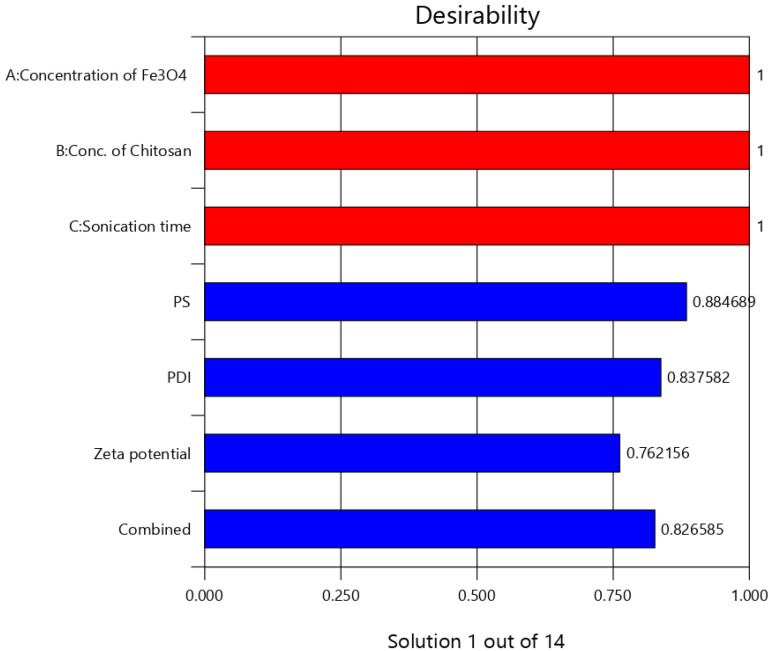
Bar chart of overall desirability.

**Figure 5:**
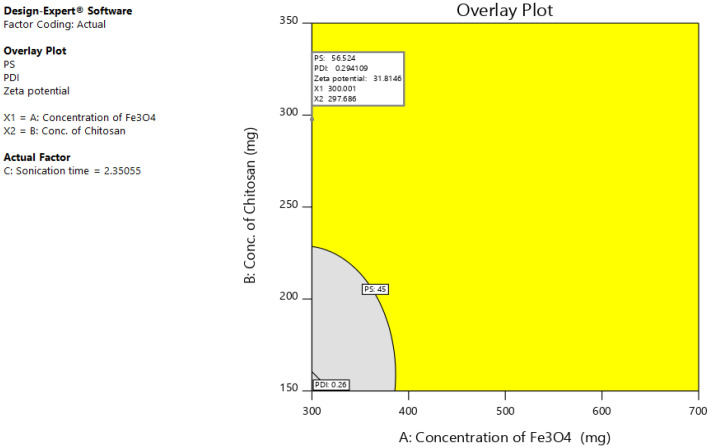
Overlay plot of the optimized formulation.

The independent effect of selected variables can be visualized and determined using RSM, by elucidating both interaction and main effect (Figure 3). To optimize the sequence of models generated, the desirability (D) function was employed. Every response was set to different criteria limits such as PS and PDI as the minimum and zeta potential as the maximum. All variables were involved in the design space for optimization. D value of 0.827 was obtained with the optimum concentrations of selected variables (Figure 4). On the basis of D function, the formulation prepared with 300 mg of Fe_3_O_4_, 297.7 mg of CS, and sonication time of 2.4 h, which can achieve the prerequisites of the target formulation. Thus, using these settings can lead to attaining minimum PS (56.5 nm) and PDI (0.3) and maximum zeta potential (31.8). By using these concentrations, O-MNP was prepared to validate the experimental and also carry out the leftover evaluation tests. Relative error was noted to be less than 2%, confirming the accuracy of the design [52,53]. The reproducibility of the design was further supported by coefficients of variation values. As required CV for all the models was found to be 10 (4.7 PS; 3.6 PDI, and 6.6 zeta potential) [54]. The overlay plot of optimized formulation and final point prediction of the design were shown in Figure 5 and Table 5.

### 3.3. PS, PDI and Surface Charge

**Figure 6:**
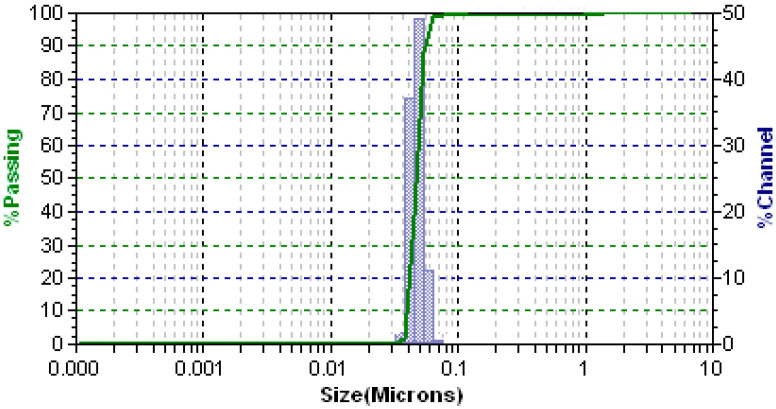
Particle size distribution of optimized magnetic nanoparticles.

PS and zeta potential are vital factors that decide the fate of nanoparticle systems in the biological environment [55,56]. Particle size distribution was found to be in the range of 35–70 nm with a narrow PDI of 0.03 ± 0.014 (Figure 6), confirming the homogeneity of the formulation. The zeta potential of the optimized formulation was found to be 31.2 ± 0.6 mV. The size and surface charge of the nanoparticles seems to be dependent on the CS concentration and agrees with an earlier study [57]. All these obtained values were following the predicted results.

### 3.4. Surface Bindingand SEM

**Figure 7:**
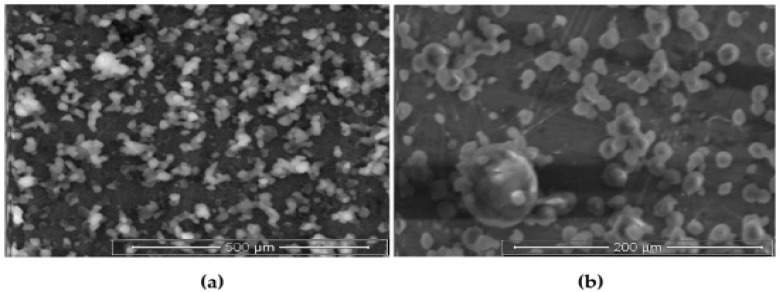
Scanning electron microscopy image of optimized magnetic nanoparticles with gemcitabine at (**a**) low (8066×) and (**b**) high magnifications (16,133×).

High surface binding of gemcitabine (91.25%) was observed and this is a result of the use of CS. However, we are unable to extrapolate the same results with Fe_3_O_4_ owing to its diverse behavior at higher concentrations [53]. The surface morphology of the O-MNP formulation was found to be almost spherical and is in nanosize as per Figure 7. Ferrous sulfate can be observed with its rough surface over the particles [40,58].

### 3.5. Measurement of Magnetization

**Figure 8:**
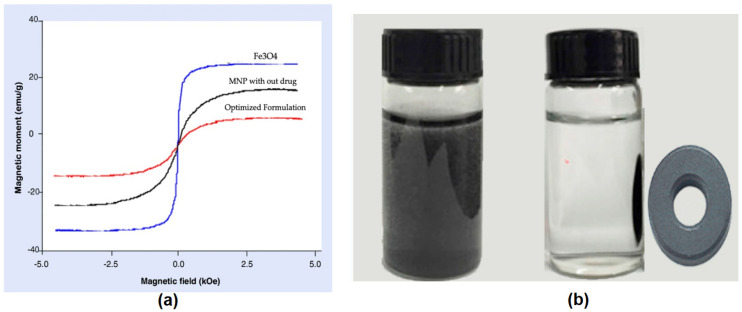
(**a**) Magnetization hysteresis loops of Fe_3_O_4_, MNPs without drugs and with an optimized formulation; (**b**) magnetic property of gemcitabine magnetic nanoparticles.

Magnetic hysteresis loops of Fe_3_O_4,_ MNP without drugs and with an optimized formulation were determined and proven as superparamagnetic materials (Figure 8a). The magnetization value of Fe_3_O_4_ and the O-MNP formulation was found to be 72.93 emu/g and 49.87 emu/g, respectively. The saturation of magnetization of O-MNP was smaller than that of colloidal Fe_3_O_4,_ but both particles had similar properties that were close to the paramagnetic behavior. The magnetic susceptibility (29 × 10^−6^) evident a clear magnetic response, moreover the nanoparticles can readily be moved and collected with an external magnetic field (Figure 8b).

### 3.6. In Vitro Drug Release

**Figure 9:**
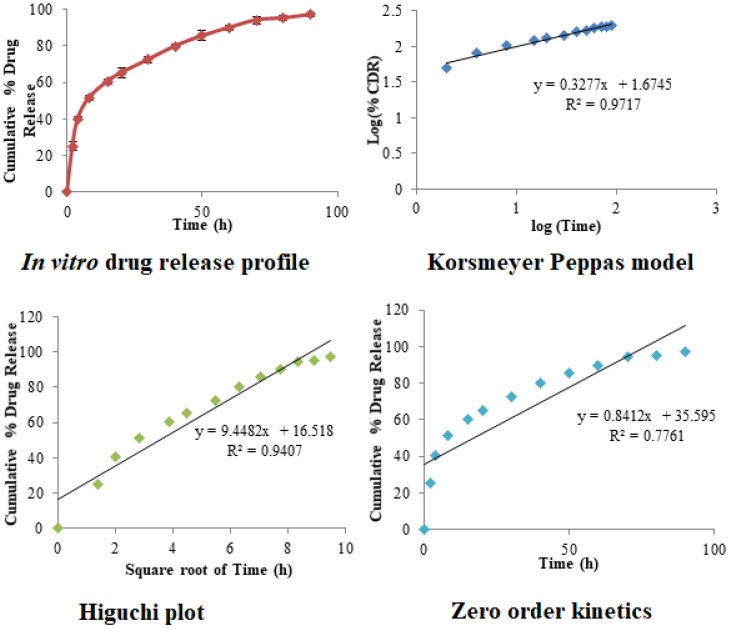
In vitro drug release profile and various kinetic models for optimized magnetic nanoparticles.

Drug release from O-MNP was demonstrated using a diffusion model. Various kinetic models [59] were applied and shown in Figure 9. Initial burst release was observed initially up to 30 h with drug release of 72.54 ± 1.95%. Consequently, a plateau was observed up to the end of 90 h. Initial burst release will help to achieve therapeutic range followed by controlled drug release over an extended period. Korsmeyer–Peppas model showed the highest R^2^ value (0.971) followed by the Higuchi model (0.941). The exponent of the Korsmeyer–Peppas model was found to be ≤0.45, which confirms the Fickian diffusion (Case I diffusional) drug release mechanism.

### 3.7. Cytotoxicity Studies

**Figure 10:**
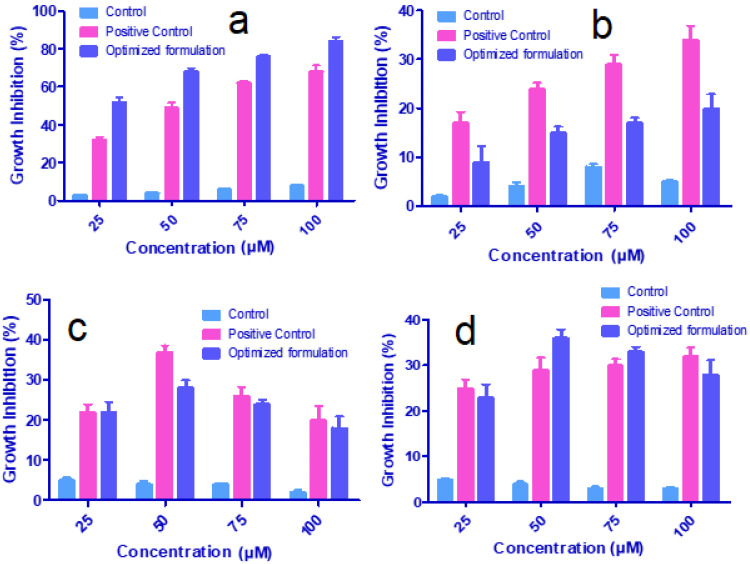
Growth inhibition (%) of control, positive control, and optimized magnetic nanoparticles with (**a**) breast cancer cell lines (MCF-7), (**b**) leukemia cancer cell lines (THP-1), (**c**) prostate cancer cell lines (PC-3), and (**d**) lung cancer cell lines (A549).

Figure 10, shows the in vitro cancer activity of O-MNP against four human cancer cell lines. Cytotoxicity was increased along with an increase in the concentration of test samples, more considerably in MCF-7 cells than other cell lines. About 84% of MCF-7 cells were killed by O-MNP at its maximum concentration (100 µM). The positive control (Epirubicin) showed only 68% of cell growth inhibition. A considerable increase in the growth inhibition was observed even with control from 25–100 µM. The cytotoxic effect of O-MNP was also observed with THP-1, PC-3, and A549 cell lines. Surprisingly, O-MNP showed less inhibition in contrast to positive control with THP-1 cells. Dose-dependent relation was observed for both the samples from 25–100 µM.

However, this relation was not evident with PC-3 cells, where the activity was increased up to 75 µM, but then declined on increasing the concentration to 100 µM. A549 cells showed quite different results, formulation showing declined activity after 75 µM, but the positive control extended its activity even beyond this concentration. From all these, we can conclude that the formulated O-MNP showed superior activity against MCF-7 cells contra to epirubicin. This can be due to the higher penetration and releasing efficacy of MNPs. Additionally, it can provide the optimum drug amount that should reach the target site. All these findings make the magnetic systems an ideal carrier to improve therapeutic efficacy. Yet, these studies should extend to in vivo models to confirm these results.

## 4. Conclusions

MNP was prepared by using a co-precipitation method and the process was optimized using Box–Behnken design. MNP has a large surface-to-volume ratio and can cause agglomeration. Initially, FTIR studies confirmed the compatibility between Gemcitabine and the selected excipients. Optimized formulation was evaluated for various in vitro parameters. The formulation was shown to have high surface binding, nanoparticle size, narrow PDI, and good zeta potential. Further SEM analysis confirmed the spherical morphology and monodispersity were evident from low PDI values. In vitro drug release pattern showed the complete release of drug in a more controlled manner up to 90 h. In vitro cytotoxicity studies on four human cancer cells confirm that O-MNP showed better growth inhibition (84%), especially against MCF-7 cells. Nevertheless, all these findings should be confirmed with in vivo cancer studies.

## Figures and Tables

**Table 1:** Experimental Plan as Per Box–Behnken design.

Factors/Independent Variables	Levels			Responses/Dependent Variables	Constraints
	−1	0	+1		
Concentration of Fe_3_O_4_ (g)-X_1_	300	500	700	Particle size (nm)	Minimum
Conc. of Chitosan (g)-X_2_	150	250	350	Polydispersity index	Minimum
Sonication time (h)-X_3_	1.5	2	2.5	Zeta potential (mV)	Maximum

**Table 2:** Experimental runs projected and their measured responses.

Run	A:Conc. of Fe_3_O_4_ (g)	B:Conc. of Chitosan (g)	C:Sonication Time (h)	Particle Size (nm)	Polydispersity Index	Zeta Potential (mV)
1	500	250	2	69	0.36	36
2	500	350	1	105	0.42	27
3	500	250	2	68	0.36	38
4	700	250	1	142	0.47	29
5	500	150	1	96	0.43	18
6	300	250	3	54	0.29	25
7	500	250	2	71	0.38	33
8	700	250	3	119	0.33	29
9	700	150	2	124	0.32	24
10	500	350	3	98	0.29	28
11	700	350	2	145	0.32	30
12	500	250	2	70	0.39	35
13	300	350	2	65	0.29	31
14	500	150	3	54	0.31	18
15	500	250	2	69	0.37	37
16	300	250	1	75	0.41	27
17	300	150	2	45	0.26	12

**Table 3:** Fit statistics for all the responses.

	Particle Size	Polydispersity Index	Zeta Potential
Std. Dev.	4.10	0.0128	1.83
Mean	86.41	0.3529	28.06
C.V. %	4.74	3.64	6.54
Sequential *p*-value	<0.0001	0.0002	<0.0001
Lack of Fit *p*-value	0.0636	0.5034	0.5600
R²	0.9826	0.9790	0.9712
Adjusted R²	0.9418	0.9520	0.9341
Predicted R²	0.8830	0.8424	0.8003
Adeq. Precision	31.552	20.4276	15.9408

**Table 4:** Coefficients table of ANOVA for all the responses.

	Intercept	A	B	C	AB	AC	BC	A²	B²	C²
Particle size	36.375	11.75	−11.625	0.25	−0.5	8.75	17.3	8.05	10.8	36.375
*p*-values	<0.0001	<0.0001	<0.0001	0.9063	0.8141	0.0037	<0.0001	0.0050	0.0010	<0.0001
Polydispersity index	0.02375	−2.95384 × 10 ^−17^	−0.06375	−0.0075	−0.005	−0.0025	−0.031	−0.0435	0.034	0.02375
*p*-values	0.0012	1.0000	<0.0001	0.2811	0.4618	0.7087	0.0017	0.0002	0.0010	0.0012
Zeta potential	2.125	5.5	−0.125	−3.25	0.5	0.25	−3.4	−8.15	−4.9	2.125
*p*-values	0.0135	<0.0001	0.8526	0.0094	0.6025	0.7930	0.0067	<0.0001	0.0009	0.0135

**Table 5:** Final point prediction of design.

Solution 1 of 12 Response	Predicted Median	95% CI Low for Mean	95% CI High for Mean	95% TI Low for 99% Pop	95% TI High for 99% Pop
Particle size	56.5295	50.1251	62.9338	32.4364	80.6225
Polydispersity index	0.2941	0.274017	0.314184	0.218547	0.369654
Zeta potential	31.8148	28.9471	34.6826	21.0263	42.6033

## Data Availability

The data presented in this study are contained within the article.
